# *N*-Glycosylation of Cholera Toxin B Subunit: Serendipity for Novel Plant-Made Vaccines?

**DOI:** 10.3389/fpls.2015.01132

**Published:** 2015-12-22

**Authors:** Nobuyuki Matoba

**Affiliations:** Department of Pharmacology and Toxicology and Owensboro Cancer Research Program of James Graham Brown Cancer Center, University of Louisville School of MedicineOwensboro, KY, USA

**Keywords:** Cholera toxin B subunit, *N*-glycosylation, plant-made pharmaceutical, subunit vaccine, C-type lectin receptors

## Abstract

The non-toxic B subunit of cholera toxin (CTB) has attracted considerable interests from vaccinologists due to strong mucosal immunomodulatory effects and potential utility as a vaccine scaffold for heterologous antigens. Along with other conventional protein expression systems, various plant species have been used as production hosts for CTB and its fusion proteins. However, it has recently become clear that the protein is *N*-glycosylated within the endoplasmic reticulum of plant cells—a eukaryotic post-translational modification that is not present in native CTB. While functionally active aglycosylated variants have been successfully engineered to circumvent potential safety and regulatory issues related to glycosylation, this modification may actually provide advantageous characteristics to the protein as a vaccine platform. Based on data from our recent studies, I discuss the unique features of *N*-glycosylated CTB produced in plants for the development of novel vaccines.

## Introduction

Cholera toxin B subunit (CTB) is a non-toxic component of cholera holotoxin, the virulence factor of *Vibrio cholerae* (Baldauf et al., 2015). The subunit non-covalently assembles into a homopentamer structure, which allows for high-affinity interaction with its receptor GM1-ganglioside present on the surface of mammalian cells. A recombinant, bacterial fermentation-derived CTB is included in an oral cholera vaccine (Dukoral®), which has been used in Sweden since 1991 and granted a marketing authorization throughout the European Union by the European Commission in 2004 (European Medicines Agency, 2014). Accordingly, CTB represents one of a few recombinant subunit vaccines currently approved for human use, and it is the only one that is capable of eliciting an effective immune response via oral delivery. Upon oral administration, CTB induces a robust antibody response in systemic and mucosal compartments, thereby neutralizing the holotoxin secreted by the bacteria. Such a strong oral immunogenicity makes CTB among the most potent mucosal immunogens described to date (Lycke, 2012), and therefore the protein provides an attractive vaccine platform for the induction of a protective antibody response to heterologous antigens. Meanwhile, recent studies have shown that CTB has unique anti-inflammatory activity against immunopathological conditions in allergy and inflammatory diseases (reviewed in Sun et al., 2010; Baldauf et al., 2015). For example, oral administration of CTB was shown to mitigate Crohn's disease in humans (Stål et al., 2010). A human 60 kD heat-shock protein (HSP60)-derived peptide, p336–351, was chemically linked to CTB, and this CTB conjugate protein (p336–351-CTB) was shown to prevent relapses of uveitis in Behcet's disease in a phase I/II clinical trial (Stanford et al., 2004). Collectively, CTB is a multifunctional mucosal immunomodulatory protein that serves not only as a cholera vaccine antigen, but also as a molecular scaffold for novel mucosal vaccines and immunotherapeutics. Numerous studies have explored such possibilities for various diseases, which are reviewed elsewhere (Baldauf et al., 2015; Stratmann, 2015).

Since the late 90's, a variety of plant species have been used to constitutively or transiently express CTB and CTB-antigen fusion proteins, including tobacco (*Nicotiana tabacum* and *N. benthamiana*), potato, rice and tomato, among others (reviewed in Baldauf et al., 2015). These studies have shown that plant-expressed CTB proteins formed pentamer structure, retained binding affinity to GM1-ganglioside and induced relevant antibody responses upon mucosal immunization. However, we and others have recently shown that plant-expressed CTB (with an exception of chloroplast-targeted expression, e.g., Daniell et al., 2001) is *N*-glycosylated within the endoplasmic reticulum (ER) of plant cells, a eukaryotic post-translational modification not present in the original protein (Mishra et al., 2006; Matoba et al., 2009; Yuki et al., 2013). While this modification does not appear to compromise CTB's principal bioactivity, i.e., mucosal immunogenicity, plant-specific glycoforms may lead to potential safety issues such as hypersensitivity or allergy (Dicker and Strasser, 2015). It should be noted that plant-specific glycosylation *per se* does not necessarily pose an additional regulatory risk in biopharmaceuticals development unless there is evidence for product-specific safety and/or efficacy issues found in preclinical or clinical studies. In fact, no major adverse event associated with plant-specific glycosylation has been reported for plant-made biopharmaceuticals that have obtained a regulatory approval for marketing or emergency use [e.g., carrot cell-produced β-glucocerebrosidase (Grabowski et al., 2014; Pastores et al., 2014) and a *N. benthamiana*-produced H5N1 avian influenza virus-like particle vaccine (Landry et al., 2010; Ward et al., 2014), respectively]. Nevertheless, glycosylation would add a regulatory complication because of glycan heterogeneity. As a consequence of these theoretical concerns, *N*-glycosylated CTB might be viewed inferior to the non-glycosylated counterpart—*unless* there is a good reason to keep the modification. Based on our recent findings, potential advantages of CTB glycosylation for vaccine development are discussed below.

## *N*-glycosylation of CTB in plants

**Figure 1:**
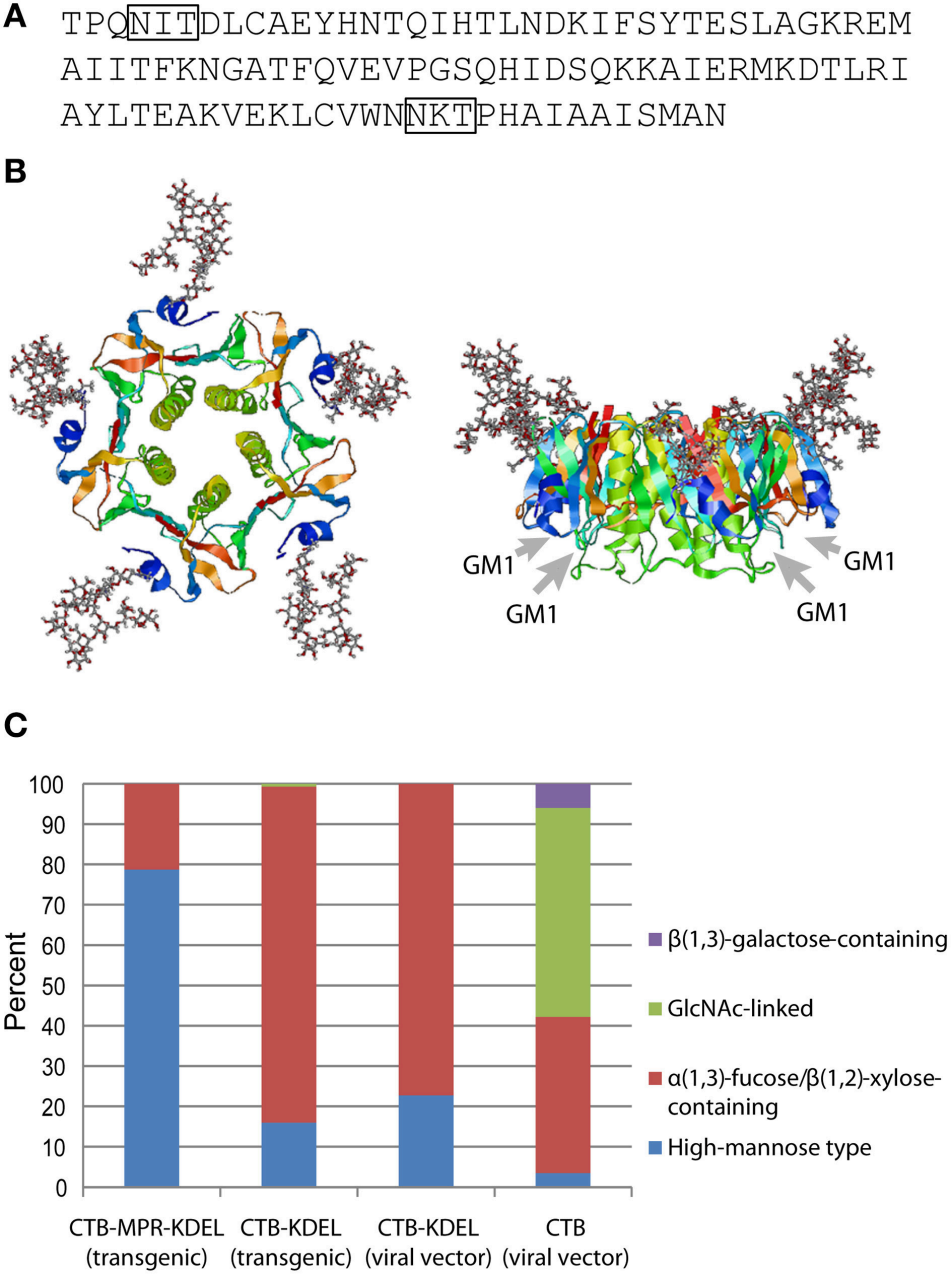
***N*-Glycosylation of CTB**. **(A)** Amino acid sequence of CTB from *V. cholerae* 569B strain (Protein Data Bank ID: 1FGB). *N*-Glycosylation sequons (Asn-X-Thr/Ser) are boxed. **(B)** Hypothetical structure images showing CTB homopentamer with high-mannose-type glycans attached to Asn4 positions. Images, top view on the left and side view on the right, were created by the Glyprot *in silico* protein glycosylation tool (http://www.glycosciences.de/modeling/glyprot/) based on the crystal structure of CTB (Protein Data Bank ID: 1FGB) and visualized by RasWin Molecular Graphics (ver. 2.7.5.2). Gray arrows show the positions of GM1-ganglioside-binding pockets. **(C)** Percent compositions of glycoforms attached to Asn4 of CTB, CTB-KDEL, and CTB-MPR-KDEL expressed in *N. benthamiana*. Data derived from Matoba et al. (2009), Hamorsky et al. (2013, 2015).

The first experimental evidence for CTB glycosylation *in planta* was reported by Mishra et al. (2006). The authors showed that CTB expressed in transgenic tobacco was modified with a ~3 kD glycan (per monomer), which was demonstrated by Schiff's test, concanavalin A binding, as well as chemical and enzymatic deglycosylation. Subsequently, Asn4 of CTB and a CTB-fusion protein were shown to be glycosylated in transgenic *N. benthamiana* (Matoba et al., 2009; Hamorsky et al., 2013) and transgenic rice (Yuki et al., 2013). Among the two potential *N*-glycosylation sites in the amino acid sequence of CTB, one at the near C-terminus (Asn90-Lys91-Thr92; Figure 1A) was not glycosylated because the sequon is immediately followed by Pro, which is known to abolish *N*-glycosylation (Jones et al., 2005). Figure 1B shows Asn4-linked glycans modeled in the context of a CTB crystal structure. It is apparent that the glycosylation site is exposed and located away from the GM1-ganglioside-binding pocket, suggesting that the oligosaccharides would not affect the protein's receptor binding affinity. This was experimentally demonstrated in our previous studies based on competitive GM1-ganglioside-capture enzyme-linked immunosorbent assays (ELISA) and surface plasmon resonance. Additionally, *N. benthamiana*-expressed Asn4-glycosylated CTB (gCTB) showed acid and thermal stabilities as well as oral vaccine efficacy for the induction of immunoglobulins (Igs) against cholera holotoxin that were comparable to those of the non-glycosylated original protein (Hamorsky et al., 2015).

Figure 1C shows the overall *N*-glycan profiles of four gCTB proteins that we have expressed in *N. benthamiana*, either constitutively via nuclear transformation or transiently using a viral vector. Among these, three contained an ER retention signal (KDEL) at the C-terminus. Unlike high-mannose-rich gCTB-MPR-KDEL expressed in transgenic *N. benthamiana*, gCTB-KDEL produced in transgenic *N. benthamiana* showed a different glycan profile, with >80% being plant-specific α(1,3)-fucose and/or β(1,2)-xylose glycoforms (Hamorsky et al., 2013). The distinct glycan profiles of these transgenic plant-expressed gCTB proteins likely reflects their difference in subcellular distribution *in planta*; although both contained a KDEL signal sequence, the fusion protein had a longer extension on the C-terminus due to the 36-amino-acid MPR domain, which might have facilitated KDEL receptor recognition for more efficient ER retention. It is of interest to note that gCTB-KDEL, when transiently overexpressed using a tobamovirus vector, showed an overall similar glycan profile to that of the same protein expressed in transgenic plants (Figure 1C), despite that the two expression systems had completely different production rate and yield; ~3 g of gCTB-KDEL were obtained per kg of leaf material in five days in the transient system (Hamorsky et al., 2015), whereas ~0.1 g of the protein were constitutively expressed per kg of transgenic leaves (Hamorsky et al., 2013). This suggests that the ER retention efficiency of gCTB-KDEL is similar regardless of the speed of protein biosynthesis. Meanwhile, gCTB devoid of KDEL showed a markedly distinct glycan profile with large fractions of complex glycoforms.

Altogether, the above findings showed the substantial heterogeneity of *N*-glycans attached to CTB expressed in plants, which in turn revealed the limitation of the KDEL-based ER retention strategy to control such heterogeneity. Cognizant of potential safety concerns and regulatory complications related to glycan heterogeneity and/or plant-specific glycoforms, we and others have developed aglycosylated CTB mutants for vaccine development; we mutated Asn4 of CTB-KDEL to Ser because the closely related *E. coli* heat-labile enterotoxin B subunit has Ser at the corresponding position (Hamorsky et al., 2013), while Yuki et al. changed Asn4 of CTB (no KDEL) to Gln (Yuki et al., 2013). Both of these CTB variants were shown in animal models to efficiently elicit cholera holotoxin-neutralizing antibodies upon oral immunization, demonstrating that Asn4 mutations did not affect the protein's vaccine efficacy. These results underscore that plant-made aglycosylated CTB variants can serve as an alternative to the bacteria-produced recombinant protein currently used in an oral cholera vaccine product.

## Potential advantages of *N*-glycans attached to CTB

Given that functionally active aglycosylated CTB variants can be produced in plants, why bother considering the protein's *N*-glycosylation any further? In this regard, recently we found an interesting function of *N*-glycosylation for transient overexpression of CTB in *N. benthamiana*. When an aglycosylated CTB variant (N4S-CTB; no C-terminal KDEL) was expressed using a plant virus vector, the protein induced strong ER stress and massive tissue damage, resulting in a poor yield (< 10 mg of GM1-ganglioside-binding CTB per kg of leaf material; Hamorsky et al., 2015). In sharp contrast, gCTB (no KDEL) did not show any significant stress response, either at gene expression (*PDI, BiP*, and *bZIP60*) or macroscopic levels. Moreover, the protein was very efficiently expressed and accumulated in a functional pentamer form in leaf tissue. The expression level reached up to 3 g of gCTB per kg of leaf biomass, which is among the highest yields for recombinant protein production in plants reported thus far (Hamorsky et al., 2015). Based on data obtained by gene expression and protein ubiquitination analyses, we concluded that the efficient “nursing” of nascent gCTB polypeptides by lectin chaperones (Molinari, 2007; Aebi, 2013) facilitated the assembly of the pentameric protein in the ER, thereby mitigating unfolded protein response that would otherwise lead to strong ER stress and tissue necrosis. Although the critical role of *N*-glycosylation in the quality control of newly synthesized proteins has been well known (Helenius and Aebi, 2004; Braakman and Bulleid, 2011), the above study highlighted the significance of such a role for the efficient bioproduction of recombinant glycoproteins in plant-based transient overexpression systems.

Thus, a proven advantage of CTB *N*-glycosylation is the significant improvement of production yield in plants. Although the aforementioned complications around glycans still need to be addressed, these issues have been under extensive investigations in recent years (Dicker and Strasser, 2015). It is expected that glycoengineering of host plants will soon generate a superior expression platform that can provide recombinant glycoproteins with more uniform, mammalian cell-like glycans (Strasser et al., 2014). In parallel, advances are being made in refining and simplifying glycan analysis technologies that can meet regulatory requirements for biopharmaceutical production (Higgins, 2010; Shubhakar et al., 2015). Given these efforts in the two front lines against glycosylation issues, it is the author's opinion that the currently perceived inferiority of *N*-glycosylated CTB is not an insurmountable challenge to overcome. Nevertheless, a higher recombinant production yield alone may not sufficiently justify the development of *N*-glycosylated CTB for pharmaceutical use when the non-glycosylated counterpart with comparable efficacy and safety profiles can be produced in a different production platform. Below two possible scenarios are discussed that could represent additional advantages of *N*-glycosylated CTB over the non-glycosylated counterpart.

### *N*-glycans may alter the B cell antigenicity profile of CTB

Glycans are generally resistant to humoral immune recognition due to poor immunogenicity (i.e., lack of T cell epitopes) and low antigenicity (i.e., high conformational flexibility; Heimburg-Molinaro et al., 2011; Peri, 2013; Amon et al., 2014). This is particularly true for “self” sugar structures that are found in humans; glycosylation of proteins with conserved mammalian sugars generally diminishes product immunogenicity, as discussed in a recent U.S. Food and Drug Administration (FDA) guidance document for immunogenicity assessment of therapeutic protein (FDA, 2014). Many enveloped viruses take advantage of this unique immunological feature of carbohydrate molecules by using envelope glycans as a “shield” to escape from humoral immunity (Vigerust and Shepherd, 2007). For example, studies have shown that influenza A viruses exploit *N*-glycans on the globular head of hemagglutinin, where the sialic acid-binding site is located, to mask the critical epitopes recognized by neutralizing antibodies (Tate et al., 2014). Human immunodeficiency virus type-1 (HIV-1) generates neutralization escape mutants in each infected individual by changing its envelope *N*-glycosylation pattern (Wei et al., 2003). Hepatitis C virus also uses the glycan shield strategy to reduce the humoral immunogenicity of envelope proteins and mask neutralizing epitopes (Helle et al., 2011). These observations point to a possibility of utilizing *N*-glycosylation to modify CTB's antigenic profile. In line with this notion, a recent study has shown that *N*-glycosylation of a malaria antigen (PfAMA1) produced in *N. benthamiana* has modified the protein's antigenicity by shielding multiple amino acid epitopes from humoral immune recognition (Boes et al., 2015).

**Figure 2:**
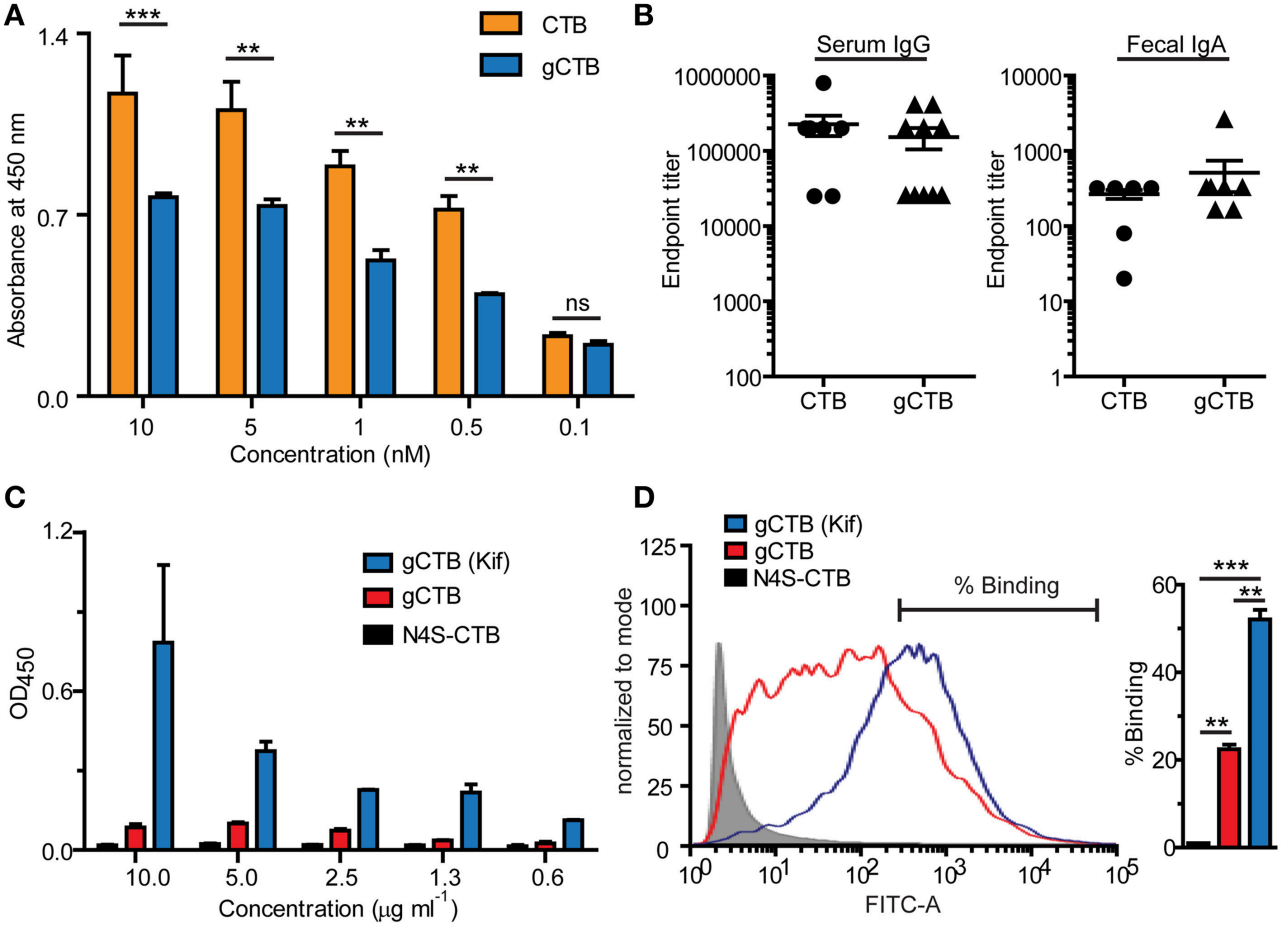
**Immune-related effects of *N*-glycans attached to CTB**. The potential impacts of Asn4-glycans on humoral immunity **(A, B)** and dendritic cell-specific intracellular adhesion molecule 3-grabbing non-integrin (DC-SIGN) binding **(C, D)** are shown. **(A)** Reactivity of a commercial polyclonal anti-CTB antibody product (List Biological Laboratories) to native CTB (Sigma-Aldrich) and Asn4-glycosylated CTB expressed in *N. benthamiana* (gCTB; no C-terminal KDEL attached). An ELISA plate was coated with GM1-ganglioside, to which varying concentrations of native CTB or gCTB were added. The receptor-bound CTB or gCTB were detected by incubation with the polyclonal antibodies followed by anti-goat IgG secondary antibodies, as described previously (Hamorsky et al., 2015). Native CTB and gCTB have a comparable affinity to GM1-ganglioside (Hamorsky et al., 2015). The anti-CTB antibodies recognized native CTB significantly better than gCTB, suggesting antigenic masking or a “glycan shield” effect by Asn4 glycans. ^**^P < 0.01; ^***^P < 0.001; Two-way repeated measures analysis of variance (ANOVA) with Bonferroni's posttest (GraphPad Prism 5). **(B)** Serum and Fecal anti-cholera holotoxin antibody titers of C57bl/6 mice orally immunized with native CTB or gCTB (3 μg per mouse, twice at a 2-week interval; graphs adapted from Hamorsky et al., 2015, under the Creative Commons Attribution License). **(C)** DC-SIGN-binding activity of gCTB and an aglycosylated plant-made CTB (N4S-CTB). An ELISA plate was coated with varying concentrations of gCTB, gCTB produced in plants treated with kifunensin (Kif) or N4S-CTB, to which a human DC-SIGN-Fc fusion (Sino Biological) was added. The bound DC-SIGN was detected with an anti-human IgG Fc secondary antibody. **(D)** gCTB's binding to cell-surface DC-SIGN. Raji cells expressing DC-SIGN were incubated with Alexa Fluor® 488-labeled N4S-CTB-KDEL, gCTB, or gCTB (Kif) at a final concentration of 10 μg/ml, and analyzed by flow cytometry. ^**^P < 0.01, ^***^P < 0.001; One-way ANOVA with Bonferroni's multiple comparison test (GraphPad Prism 5). Graphs adapted from Hamorsky et al. (2015), under the Creative Commons Attribution License.

Figure 2A shows the reactivity of a commercial anti-CTB antiserum to varying concentrations of gCTB and the bacteria-produced non-glycosylated counterpart. The results clearly show the masking of a significant portion of CTB's surface epitopes that are recognized by the polyclonal antibodies, illustrating the alteration of the protein's antigenicity by Asn4-attached glycans. It is noteworthy that, despite such an antigenic masking effect, gCTB still raised comparable anti-cholera holotoxin IgA and IgG responses as native CTB upon oral administration in mice (Figure 2B; Hamorsky et al., 2015). These results indicate that Asn4-linked glycans modify the B cell antigenicity profile of CTB without affecting the protein's overall immunogenicity. Accordingly, one testable hypothesis based on these findings is that the glycans may redirect antibodies to recognize CTB's structural domains that are away from the glycosylation site, such as the foreign antigen moiety in the case of CTB-antigen fusions. We have previously observed that *N. benthamiana*-expressed CTB-MPR, an Asn4-glycosylated CTB-fusion vaccine candidate against HIV-1, could generate a measurable vaginal IgA response to the HIV-1 antigen in intranasally immunized mice, which seemed to be more effective than that induced by *E. coli*-derived CTB-MPR although immunization regimens and immunogen qualities in those studies were not comparable (Matoba et al., 2004, 2006, 2009). If this observation is confirmed in a side-by-side comparison study, it will provide an important implication for CTB fusion-based vaccine development because the CTB domain tends to be more immunodominant than bystander antigens fused to the scaffold (Matoba et al., 2006). Hence, *N*-glycosylated CTB may serve as a superior vaccine platform to the non-glycosylated counterpart for the induction of a better antibody response to genetically or chemically fused antigens.

### *N*-glycans may enhance the antigen-targeting ability of CTB via interaction with C-type lectin receptors

Complex sugars present on microorganisms, cell surfaces and glycoconjugates have a capability to elicit unique signals in the immune system by interacting with C-type lectin receptors. These carbohydrate-binding receptors are abundantly expressed on innate immune cell membranes, most notably antigen presenting cells such as dendritic cells and macrophages (Drickamer and Taylor, 2015). Since C-type lectin receptors are endocytic, glycosylated antigens are internalized after binding to the receptors and subsequently presented on major histocompatibility complex (MHC) class I and II molecules. Antigen presenting cells can then activate effector or regulatory T cell responses in cooperation with other co-stimulatory signals. An early study has shown that mannosylated peptides and proteins were efficiently taken up by dendritic cells via mannose receptors, a type of C-type lectin receptors, resulting in 200–10,000 times more efficient antigen presentation to T cells than non-mannosylated counterparts (Tan et al., 1997). Given this, a number of studies have attempted to exploit C-type lectin receptors to efficiently deliver and present antigens to T cells (Apostolopoulos et al., 2013; Lepenies et al., 2013; van Kooyk et al., 2013; Sedaghat et al., 2014). For instance, oligomannose-coated liposome was shown to be capable of delivering encapsulated protein antigens to the MHC class I and class II pathways in antigen presenting cells and thereby generating antigen-specific cytotoxic T cells and type 1 helper T cells (Kojima et al., 2013). These findings provide an implication for the potential use of *N*-glycosylated CTB.

Among different C-type lectin receptors expressed on dendritic cells, dendritic cell-specific intracellular adhesion molecule 3-grabbing non-integrin (DC-SIGN) is a major receptor that recognizes mannose-containing glycans. We tested if gCTB could bind to DC-SIGN using ELISA and flow cytometry. The results demonstrated that gCTB is capable of binding to recombinant and cell surface-expressed DC-SIGN (Figures 2C,D; Hamorsky et al., 2015). Notably, gCTB's binding affinity to DC-SIGN was significantly enhanced to a nanomolar level when the protein was produced in plants treated with the class I α-mannosidase inhibitor kifunensin, which restricts *N*-glycans to be high-mannose types (Figures 2C,D; Hamorsky et al., 2015). Such a high affinity to DC-SIGN is considered sufficient for dendritic cell targeting, internalization and cross presentation (Srinivas et al., 2007; Singh et al., 2009). Taken together, these findings lead to another testable hypothesis that *N*-glycosylation may enhance the antigen-targeting capabilities of CTB fusion proteins via DC-SIGN and other C-type lectin receptors. Particularly, the ability of C-type lectin receptors to cross-present antigens on the MHC class I molecule will broaden the potential utility of gCTB for vaccine development.

## Concluding remarks

The above two proposed scenarios highlight how *N*-glycosylation of CTB may facilitate the protein's utility as a vaccine scaffold. Glycoengineering of *N*-glycans by genetic or chemical approaches may enhance such potentials, especially by focusing on high-mannose-type glycans since these glycoforms *per se* are generally not immunogenic in mammalians. Hence, for mucosal antibody induction these glycans may effectively guide B cells to recognize critical epitopes of CTB-antigen fusion proteins. On the other hand, high-mannose-glycans may facilitate the targeting of CTB-antigen fusion to C-type lectin receptors on antigen presenting cells, providing a new strategy to induce antigen-specific T cell responses. However, an important question remains to be addressed for the C-type lectin-targeting strategy; that is, whether glycosylation of CTB may or may not modify the protein's intrinsic immunomodulatory activity. As described above, CTB was shown to exhibit anti-inflammatory and immunosuppressive activities under certain conditions. Depending on how antigen presenting cells respond upon stimulation with *N*-glycosylated CTB, vaccine development based on the glycosylated molecular scaffold should be aimed at either effector (e.g., for cancer and infectious diseases) or regulatory (e.g., for allergy and autoimmune disorders) T cell responses, perhaps in combination with appropriate co-stimulatory molecules such as cytokines and toll-like receptor ligands. Because C-type lectin-mediated signaling is not fully understood (Drickamer and Taylor, 2015), the above question needs to be carefully addressed for each vaccine construct. Immunization experiments using *N*-glycosylated CTB antigens and corresponding non-glycosylated counterparts will be particularly useful in addressing these questions. Regardless of how *N*-glycosylated CTB instructs the immune system to respond, the protein seems to open new avenues for subunit vaccine development; the bottom line is that *N*-glycosylated CTB is highly bioproducible in plants, a trait that can maximize the long-discussed advantages of plant-made vaccines.

## Author contributions

NM solely conceived and wrote the manuscript.

### Conflict of interest statement

The author declares that the research was conducted in the absence of any commercial or financial relationships that could be construed as a potential conflict of interest. The author has filed a patent application concerning the concepts described in this manuscript (U.S. Patent Application serial no. 14/005,388).
